# The Meaning of Age or Generational Differences in U.S. Political Values and Priorities

**DOI:** 10.1093/geroni/igaa057.2368

**Published:** 2020-12-16

**Authors:** Judith Gonyea, Robert Hudson

**Affiliations:** Boston University, Boston, Massachusetts, United States

## Abstract

Nations globally are facing the fiscal consequences of being aging societies, including the redistribution of wealth resources across sectors that influence generational relations (i.e., healthcare, education, public pensions). Political differences or clashes between youth and older adults is not a new phenomenon. However, questions are being raised about whether current political systems, governing structures, and social trends are eroding generational solidarity which traditionally has a role in promoting equity and protecting vulnerable individuals from rapid social change. Reflecting on the 2020 national election results and political opinion surveys, we explore the meaning of age or generational differences in political attitudes in an increasingly partisan society. We suggest that the use of a generational location or habitus lens, which focuses on the distinct sociohistorical realities (i.e., different reference points, systems of aspiration, sets of anxieties) that shape age groups and their interrelations, may offer insights into current political debates and divides.

